# High tumor mutational burden and *PIK3CA* mutations correlate with poor Merkel cell carcinoma–specific survival

**DOI:** 10.1172/jci.insight.197108

**Published:** 2026-04-22

**Authors:** Matheus Lobo, Furkan Bahar, Julia L. Schnabel, Joao P. Duprat Neto, Aniket Shetty, Karam Khaddour, Manisha Thakuria, Ann W. Silk, James A. DeCaprio

**Affiliations:** 1 Department of Medicine, Brigham and Women’s Hospital, Harvard Medical School, Boston, Massachusetts, USA.; 2 Department of Medical Oncology, Dana-Farber Cancer Institute, Boston, Massachusetts, USA.; 3 Department of Cutaneous Oncology, A.C. Camargo Cancer Center, Sao Paulo, Brazil.; 4 Program in Virology, Graduate School of Arts and Sciences, Harvard University, Cambridge, Massachusetts, USA.; 5 Center for Patient Derived Models, Dana-Farber Cancer Institute, Boston, Massachusetts, USA.; 6 Harvard Medical School, Boston, Massachusetts, USA.; 7 Dana-Farber/Brigham Cancer Center Merkel Cell Carcinoma Center of Excellence, Boston, Massachusetts, USA.; 8 Department of Dermatology, Brigham and Women’s Hospital, Harvard Medical School, Boston, Massachusetts, USA.

**Keywords:** Dermatology, Oncology, Virology, Molecular pathology, Oncogenes, Skin cancer

## Abstract

Merkel cell carcinoma (MCC) is a neuroendocrine carcinoma of the skin characterized by poor prognosis. This study aimed to explore the relationship between genetic alterations, tumor mutational burden (TMB), and MCC-specific survival (MCC-SS) in patients who underwent genomic profiling of tumors with OncoPanel. Univariate and multivariable analysis were used to assess the impact of genetic alterations on MCC-SS. Of the 188 identified patients, 164 were included in the analysis. The cohort had a mean age of 72.4 years (SD = 11.03), including 61.6% male. The median TMB was 5.32 (IQR = 3.04–25.53). Kaplan-Meier curves by high versus low TMB were significantly different (log-rank test, *P* = 0.017). *PIK3CA* (adjusted *P* = 0.003), *SETBP1* (adjusted *P* = 0.002), *KDR* (adjusted *P* = 0.028), and *RET* (adjusted *P* = 0.033) were selected for multivariable analysis. In the multivariable regressions, only *PIK3CA* (HR = 2.07 [95% CI, 1.10–3.88]; *P* = 0.024) remained significant. *PIK3CA* remained significant across prespecified sensitivity analyses. In this study, high TMB and *PIK3CA* alterations were associated with poor MCC-SS. Identifying a higher-risk subgroup may inform risk stratification and motivate further evaluation of PI3K pathway targeting in future studies.

## Abstract 2

Merkel cell carcinoma (MCC) is an aggressive rare cancer of the skin. High tumor mutational burden and PIK3CA alterations were associated with worse MCC-specific survival.

## Introduction

Merkel cell carcinoma (MCC) is a rare but increasingly prevalent form of skin cancer characterized by its aggressive nature and poor prognosis. The incidence of MCC has been rising, posing substantial challenges for early diagnosis and effective management. This difficulty is frequently compounded by nonspecific clinical presentation, which can lead to delayed detection and treatment. Even in its earliest stages, MCC is associated with high rates of recurrence and mortality, underscoring the urgent need for reliable prognostic factors and tools to guide clinical decision-making (1–3).

MCC predominantly affects the elderly population, with the average age of diagnosis being over 70 years (1). The main risk factors are ultraviolet radiation exposure and Merkel cell polyomavirus (MCPyV) infection. Discovered in 2008, MCPyV is attributed as the cause for 80% of MCC cases, with clonal integration of the viral DNA into the tumor genome. MCPyV status has been considered to be a prognostic factor in several prior studies (4–8). Patients with virus-positive MCC generally exhibit better survival rates compared with those with virus-negative MCC (8). However, the methods for assessing viral status vary widely across studies, leading to inconsistent results. Techniques for determining viral status include IHC, PCR, and serologic tests (AMERK) which differ in sensitivity and specificity, complicating the interpretation of viral status (7). Studies have indicated that patients with high tumor mutational burden (TMB) are often virus-negative, while those with low TMB tend to be virus positive. Despite these findings, defining a clear cutoff for TMB in MCC has not been resolved in the literature.

The current staging of MCC is based on the eighth edition of the American Joint Committee on Cancer (AJCC) guidelines (1) and considers the size of the primary tumor and the presence of nodal and distant metastases. Although well established, the AJCC staging system has limitations in predicting prognosis, with a 5-year overall survival rate of around 51% for patients with early-stage disease (3). This highlights the need for more comprehensive tools that can provide a deeper understanding of the disease’s progression and potential outcomes.

Genomic evaluation offers a promising approach to identifying biomarkers that are linked to survival or treatment response. The OncoPanel is a genomic hybrid-capture sequencing approach that determines TMB and alterations in a set of 447 cancer-associated genes (9). While genetic correlations with higher risk are well established in other tumors, such as breast cancer and melanoma, more research is needed to understand these correlations in MCC (10, 11). This study aims to explore the relationship between genetic alterations, TMB and MCC-specific survival using data from patients with MCC treated at a referral center.

## Results

We identified 188 patients with MCC who underwent OncoPanel testing in the Dana-Farber MCC database. After excluding 24 patients due to OncoPanel testing of non-Merkel cell tumors or insufficient treatment and follow-up data, 164 patients were included in the study. The clinical characteristics are detailed in Table 1. RNA-seq data was available for 47 (28.7%) patients and was used to analyze for the presence of MCPyV.

The cohort had a mean age of 72.4 years (SD = 11.03), with 101 (61.6%) males. Autoimmune diseases were present in 17 (10.4%) patients, other primary tumors in 78 (47.6%), and immunosuppression in 14 (8.5%). Following restaging per the AJCC Eighth Edition (1), 45 (27.4%) patients were stage I/II, 89 (54.3%) stage III, and 30 (18.3%) stage IV at the time of OncoPanel biopsy. The median follow-up was 49.5 months (4.0–213.0).

The median TMB was 5.32 (IQR = 3.04–25.53); 3.04 (IQR = 2.28–4.56) in low TMB (≤6.0) patients, and 26.23 (IQR = 14.64–34.63) in high TMB (>6.0) patients. There were statistically significant differences between low and high TMB groups regarding the presence of other primary tumors (*P* = 0.003) and the viral status (*P* < 0.001). Fifty-eight patients (35.4%) underwent immunotherapy treatment following the OncoPanel biopsy.

**Figure 1:**
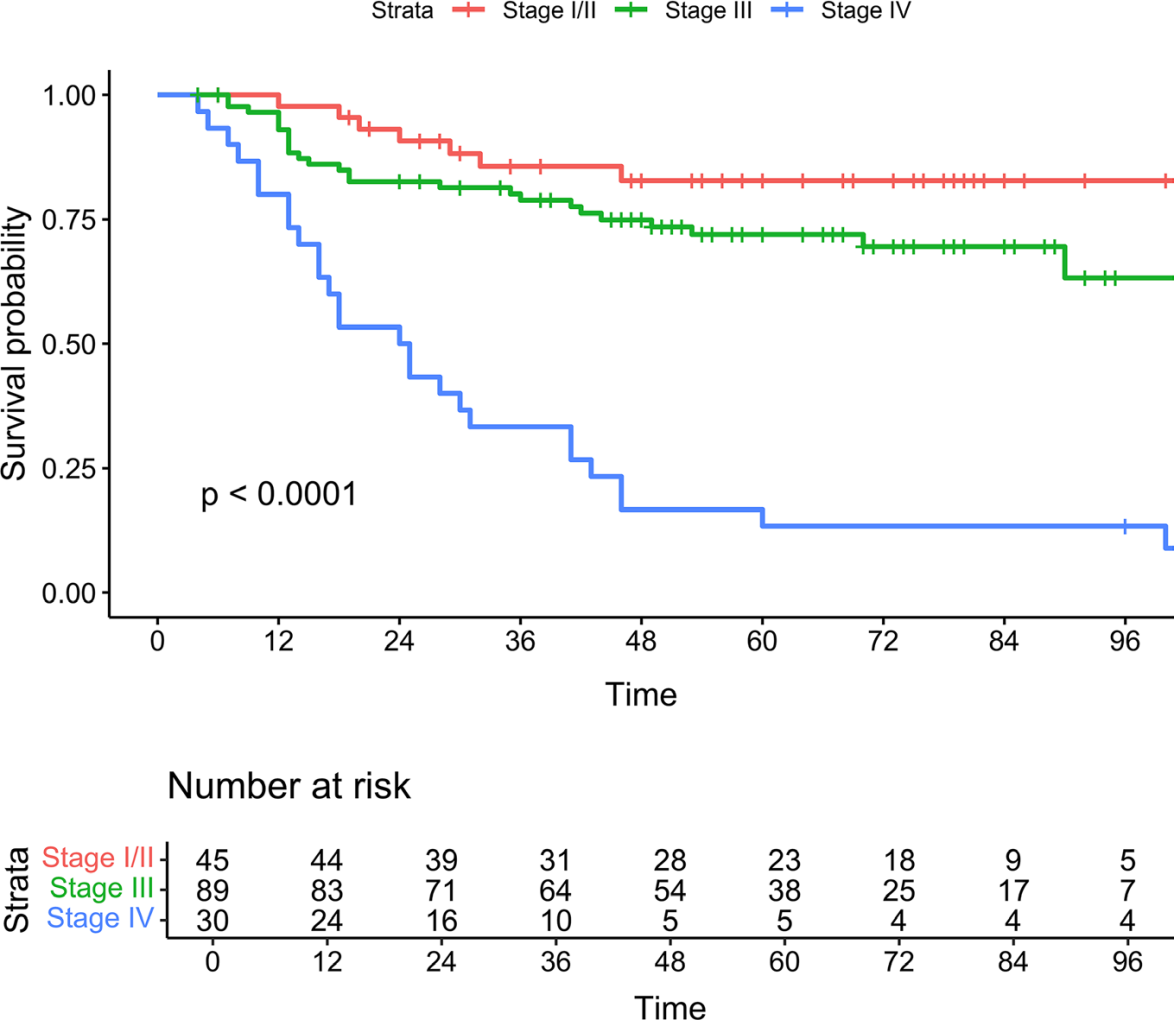
Kaplan-Meier curves for MCC-specific survival by stage according to AJCC Eighth Edition. Log-rank, *P* < 0.001.

**Figure 2:**
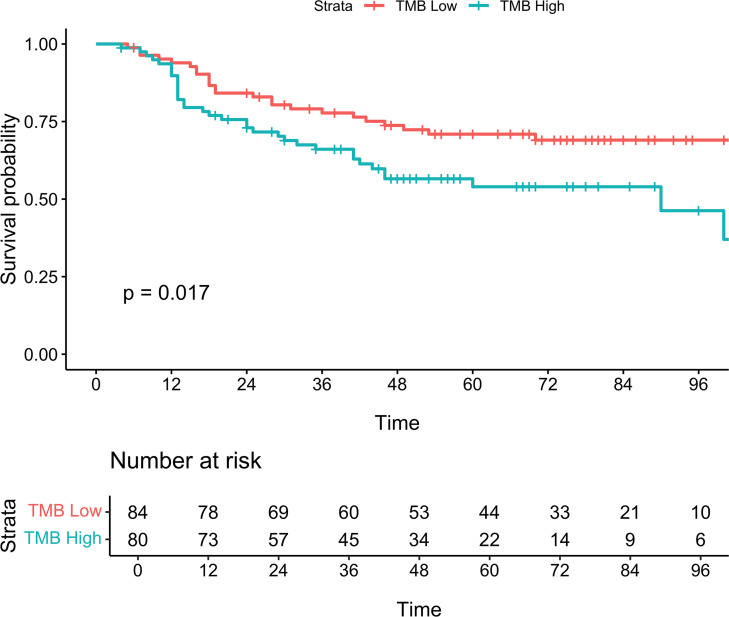
Kaplan-Meier curves for MCC-specific survival by tumor mutational burden (TMB) high (> 6.0 mut/mB) and low (≤ 6.0 mut/mB). Log-rank, *P* = 0.017.

**Figure 3:**
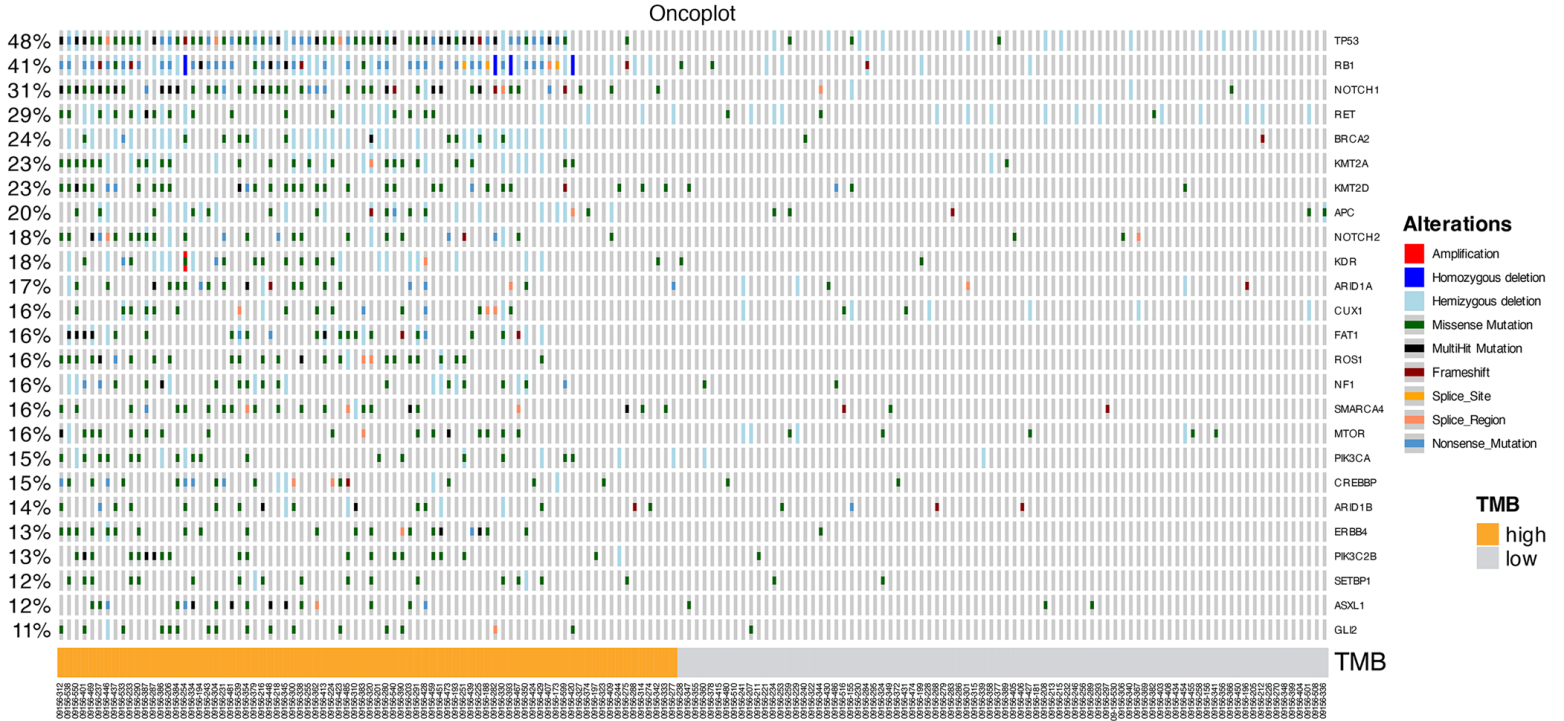
Oncoplot. Columns represent the patient’s samples, and the rows represent the top 25 gene mutations. Frequencies of gene alterations are represented in the left column. Patient’s samples are sorted by descending TMB.

Kaplan-Meier curves (Figure 1) demonstrated significant differences (log-rank test, *P* < 0.0001) in MCC-specific survival (MCC-SS) by stage. Similarly, Kaplan-Meier curves by high versus low TMB (Figure 2), were significantly different (log-rank test, *P* = 0.017) for MCC-SS. An oncoplot (Figure 3) illustrates gene alterations and TMB in all patient samples sorted by descending TMB. The most frequently altered genes were *TP53* (48%), *RB1* (41%), and *NOTCH1* (31%).

Univariate analysis identified associations between gene alterations and MCC-SS (Supplemental Table 1; supplemental material available online with this article; https://doi.org/10.1172/jci.insight.197108DS1). After correction using the Benjamini-Hochberg method, *PIK3CA* (adjusted *P* = 0.003), *SETBP1* (adjusted *P* = 0.002), *KDR* (adjusted *P* = 0.028), and *RET* (adjusted *P* = 0.033) were selected for multivariable analysis. In this cohort, 61 events occurred, prompting individual multivariable Cox regressions for the 4 genes selected. The multivariable models included the selected gene, staging, TMB, use of immunotherapy, and age. In the multivariable regressions, *PIK3CA* (HR = 2.07 [95% CI, 1.10–3.88]; *P* = 0.024) was significant, while *RET* (HR = 1.61 [95% CI, 0.94–2.74]; *P* = 0.08), *SETBP1* (HR = 1.28 [95% CI, 0.63–2.60]; *P* = 0.50), and *KDR* (HR = 1.37 [95% CI, 0.69–2.71]; *P* = 0.37) were not. Table 2 summarizes findings from univariate and multivariable analyses. The complete multivariable regressions can be found at Supplemental Materials 2–5.

Since patients with high TMB had poorer prognoses and a higher frequency of mutations, a multivariable analysis was performed within the high-TMB population, and *PIK3CA* status remained a significant predictor even in this subgroup (HR = 2.52 [95% CI, 1.20–5.27]; *P* = 0.014). The clinical characteristics of the high-TMB cohort, stratified by *PIK3CA* status, are available in Supplemental Material 6. Furthermore, 3 additional sensitivity analyses confirmed the robustness of these findings. First, the association persisted when using the Fine-Gray methodology for competing risks (HR = 2.12 [95% CI, 1.06–4.26]; *P* = 0.035). Second, a subgroup analysis excluding the immunotherapy variable, which was designed to mitigate potential immortal time bias, also identified *PIK3CA* status as statistically significant (HR = 2.43 [95%CI 1.31–4.54]; *P* = 0.005) (Table 3). Finally, when also restricting the analysis to patients with stage I–III disease, *PIK3CA* status remained significantly associated with MCC-SS after adjusting for stage (HR = 3.03 [95% CI, 1.22–7.51]; *P* = 0.017). No multicollinearity was observed in the multivariable models for either the entire cohort or the subgroup analyses. A summary of the subgroup analyses findings can be found in Supplemental Material 7.

**Figure 4:**
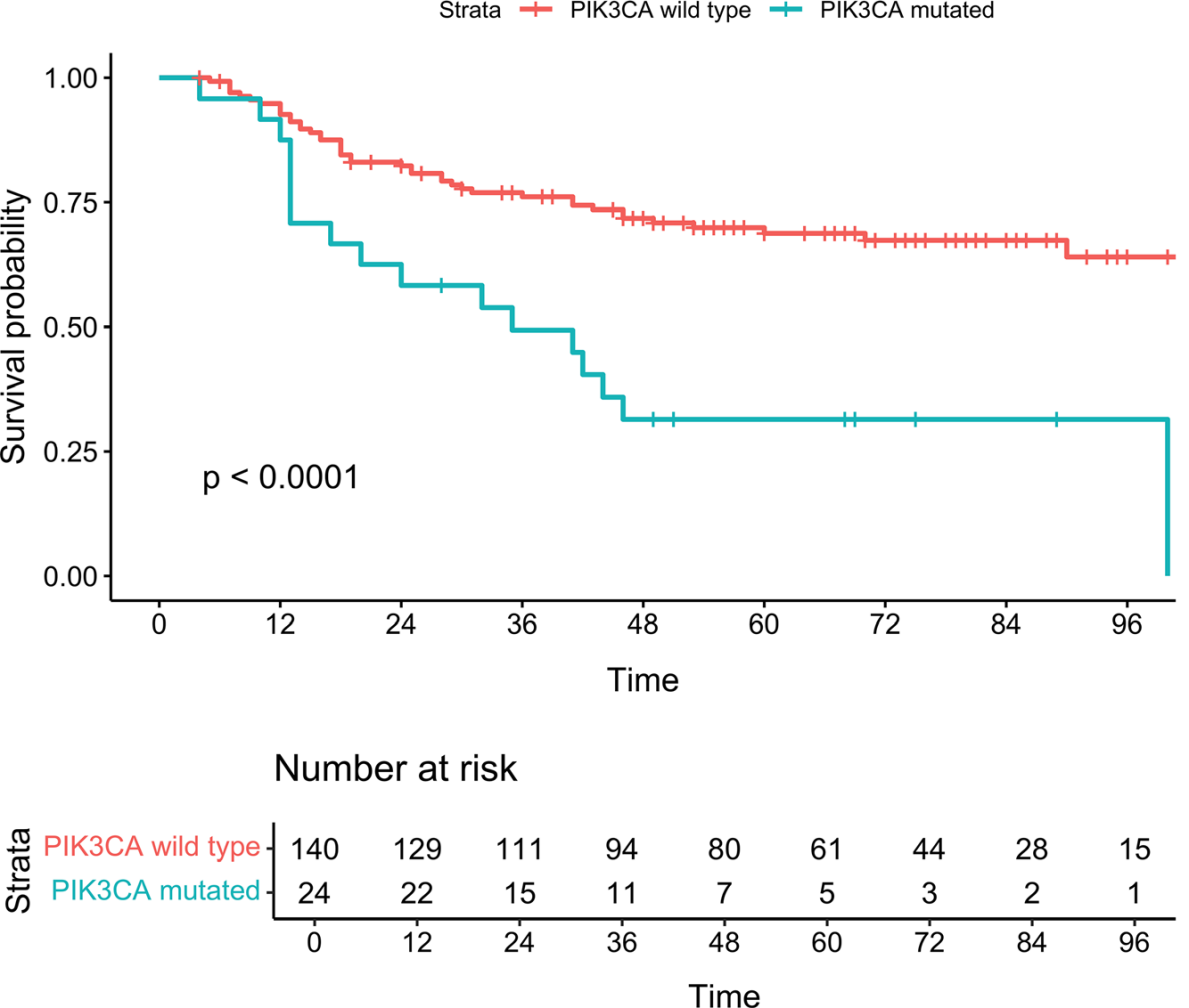
Kaplan-Meier curves for MCC-specific survival in the full cohort. Log-rank, *P* < 0.0001.

**Figure 5:**
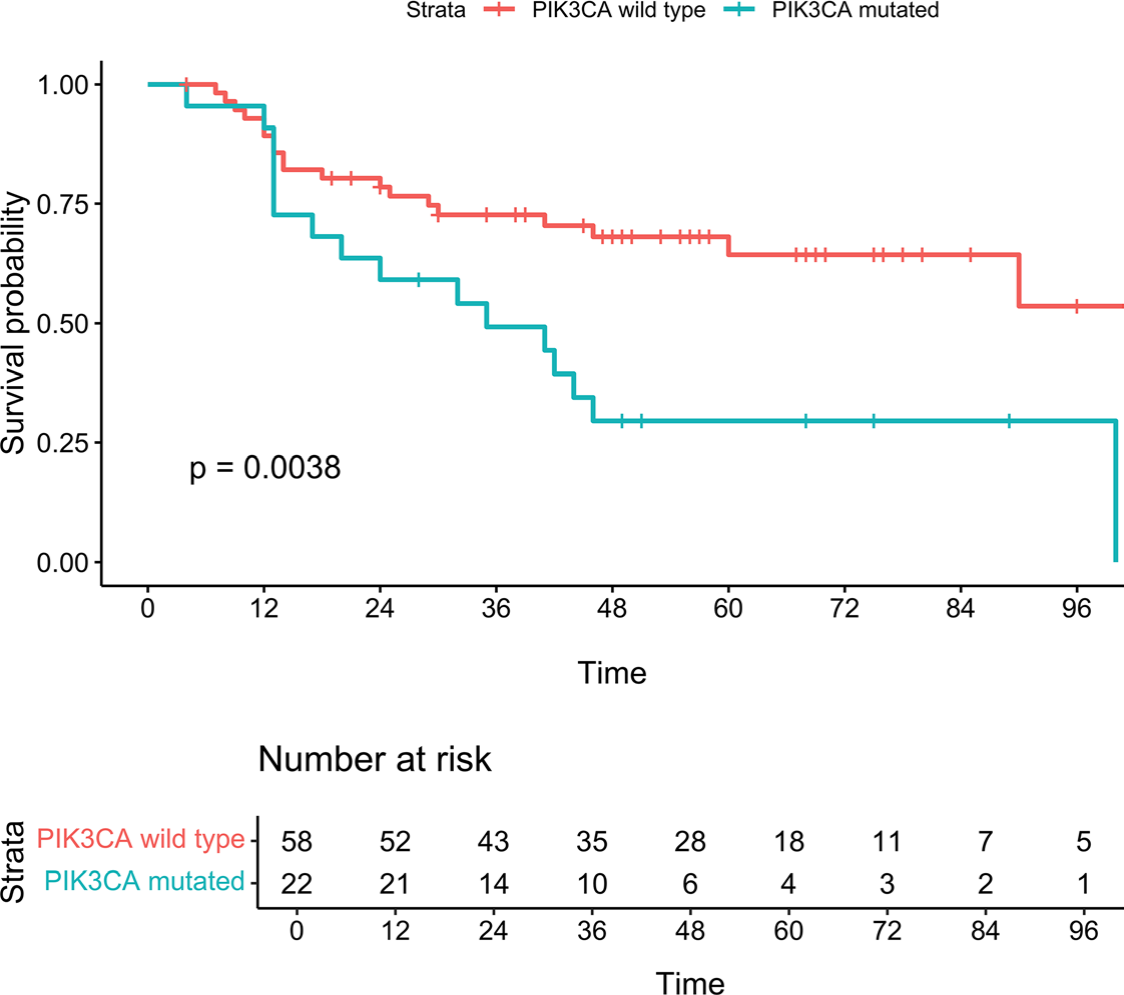
Kaplan-Meier curves for MCC-specific survival in the high TMB cohort (TMB > 6.0 mut/mB). Log-rank, *P* = 0.0038.

**Figure 6:**
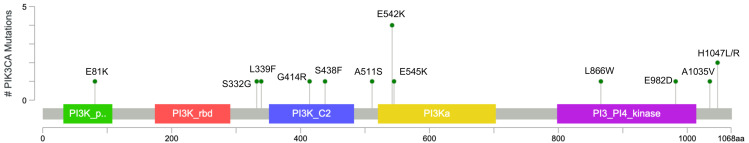
Lollipop plot showing the *PIK3CA* gene structure and illustrating missense mutation locations.

Kaplan-Meier curves for *PIK3CA* mutations showed significant differences in MCC-SS between mutated and WT patients, both in the entire cohort (Figure 4; Log-rank *P* < 0.001) and high TMB subgroup (Figure 5; Log-rank *P* = 0.004). *PIK3CA* mutations were found in 24 (14.6%) patients, with 15 missense mutations only (62.5%), 8 hemizygous deletions only (33.3%), and 1 (4.2%) presenting with both missense and hemizygous deletion. The type of alteration (missense versus hemizygous deletion) did not significantly affect MCC-SS (*P* = 0.58). Figure 6 depicts a lollipop plot illustrating the *PIK3CA* gene structure and point mutations. Eight of the 16 missense mutations were in recurrent hotspots: 1 at E545K, 2 at E542K, 4 at H1047L/R, and 1 at E81K.

## Discussion

This study primarily focuses on integrating genomic and clinical data into the analysis of patients with MCC. The assessment of the genomic profile enabled the identification of mutations associated with poorer prognosis and the evaluation of TMB in a cohort of 164 patients treated at a single institution. RNA-seq provides an orthogonal approach to viral assignment and was available in a subset of cases compared with PCR detection of integrated viral DNA and IHC staining for the large T antigen (12, 13). In our study, 20 (87%) of the 23 low TMB patients were virus positive, and 20 (83.3%) of the 24 high TMB were virus negative, reinforcing the correlation between viral status and TMB.

In this study, the high and low mutational burden subgroups were comparable except for other primary tumors and viral status. Some studies in the literature report poorer prognosis for patients with high mutational burden, using various cutoff levels. In a biomarker analysis of a phase II clinical trial, patients were categorized with a cutoff of 2.0 mut/mB based on the median, showing better survival outcomes for patients with low mutational burden (14). A recent publication reported that 82 (26.2%) of the patients with MCC had high TMB with a cutoff of 10 mut/mB (15). Notably, the TMB value of 6.0 mut/mB in our study was close to the median mutational burden (5.32), allowing for a more comprehensive analysis of survival outcomes.

In a small subset of patients, oncogenic activating mutations, including *PIK3CA*, were associated with disease-specific survival in virus-positive tumors (8). Our study shows a significant difference in survival of patients with *PIK3CA* mutation compared with WT individuals, with this effect also seen after adjustment for age, stage, immunotherapy use, and TMB. *PIK3CA* mutations are commonly associated with activation of the PI3K/AKT pathway and have 2 frequent hotspots, one in the helical domain (exon 9) and the other in the kinase domain (exon 20). These hotspots have been well described in a clinical trial of targeted therapy for breast cancer, with common point substitution mutations at residues E545, E542, and H1047 (16). Previous reports have identified 11% of *PIK3CA* mutations in patients with MCC, with mutations specifically in E545 and E542 regions, observed in other studies (17). A cross-sectional study found that 3.8% of a MCC cohort had *PIK3CA* mutation, with the majority being TMB high (15). Our study found that 14.6% of patients harbored *PIK3CA* mutations, particularly prevalent in the high TMB group, in which 22 of 80 patients (27.5%) were mutated. Also, of the 16 missense mutations observed, 50% were related to hotspots previously described in other cancer studies.

The PI3K/AKT/mTOR signaling pathway is recognized as a critical oncogenic pathway involved in various neoplasms, being altered in up to 40% of breast cancers and 32% of colorectal cancers (18, 19). PI3K heterodimer contains 2 subunits: p110α (the catalytic subunit) and p85 (the regulatory subunit). The *PIK3CA* gene encodes the p110α subunit, which catalyzes the conversion of PIP2 to PIP3. PIP3 subsequently recruits AKT to the cell membrane, together with additional factors including PDK1. AKT regulates several intracellular processes, including cell survival and metabolism, and requires full activation through phosphorylation at threonine 308 (pAKT T308) by PDK1, followed by further phosphorylation at serine 473 (pAKT S473) by mTORC2. The activation of this pathway has been previously described in MCC. The pathway activation in MCC was demonstrated by measuring the biomarker pAKT T308, stained as strong or very strong in 88% of 41 patients in IHC (20). Furthermore, it was present in both virus-positive and virus-negative patients, showing that there are potentially multiple mechanisms of activating the pathway.

Some studies have assessed the use of PI3K/AKT/mTOR pathway inhibitors alone or in combination in different cancers (21–23). Two phase 3 studies showed the benefit of PI3K pathway inhibitors (alpelisib and capvasertib) against progression or death in patients with breast cancer (16, 24). In vitro studies have demonstrated that MCC cell lines are sensitive to PI3K/AKT inhibitors, and a case report showed promising results with idelasib (PI3K inhibitor) in a patient with metastatic MCC (25).

Some limitations of this study are the inclusion of patients at different stages, the retrospective nature of the data, and the use of immunotherapy in multiple contexts. Because OncoPanel was not routinely performed for all patients with MCC, there is a potential selection bias with more advanced cases included in the study. One study highlights potential sources of bias in data analysis with genomic testing in cancer, such as immortal time bias (patients with aggressive disease who die before testing) and temporal selection bias (patients who tend to be tested when they have a worse clinical course). These biases should be considered in the interpretation of this study’s results, especially for MCC, in which some patients may have rapid progression (26, 27).

By showing that patients with high TMB have worse MCC-SS, we demonstrated that measuring TMB can aid in initial assessment and prognostication, potentially influencing decisions regarding treatment and early intervention in MCC management. Patients with *PIK3CA* mutations had poorer prognoses, even when considering the high TMB group only, highlighting the need for additional study of this potential risk group. The combination of these results, along with the identification of mutational hotspots, warrants the continued investigation of PI3K pathway inhibitor treatments in patients with advanced MCC with detected *PIK3CA* mutations.

## Methods

### Sex as a biological variable.

Our study examined male and female patients, and similar findings are reported for both sexes. To be included in the study, patients had to meet the following criteria: consent for participation in the repository, availability of sufficient OncoPanel data, and follow-up at the Dana-Farber Cancer Institute. Clinical and molecular characteristics were independently reviewed by 2 investigators and combined for subsequent statistical analysis.

### Variables.

The included variables were accessed via the electronic medical record through the EPIC portal. The list of variables and their descriptions were as follows: age (years), sex (male/female), autoimmune disease (yes/no), presence of another primary cancer (yes/no), staging according to AJCC Eighth Edition (I/II, III, or IV), TMB (continuous, in mutations/megabase [mut/mB]), and biopsy year (2013–2016 or 2017–2022). Immunotherapy was considered a binary variable, and it was included if given in either the adjuvant or metastatic setting. The selected cutoff for TMB high or low was 6.0 mut/mB, defined by a previous study (28).

The primary outcome was MCC-SS, defined as the time (in months) from diagnosis to death due to MCC or to the last follow-up. Patients who did not have an event were censored. Survival data were visualized using the Kaplan-Meier estimator, and differences between curves were assessed by the log-rank test.

### Genomic analysis.

OncoPanel was performed at the Center for Advanced Molecular Diagnostics, a CLIA-certified laboratory in the Department of Pathology at Brigham and Women’s Hospital. Genetic alterations identified through the OncoPanel, which assessed a preestablished mutations set, were evaluated for their associations with MCC-SS. Mutations were visualized using an oncoplot, displaying the frequency and clinical relevance of the identified genetic variants, and sorted by TMB. All types of mutations from the top 25 genes were included in the oncoplot and analysis, except for the less than 8 copy number gains, due to their lower biological meaning and frequent occurrence in tumor and nontumor cells. The lollipop plots were generated using the MutationMapper tool available through cBioPortal for Cancer Genomics (29, 30).

### RNA-seq.

Methods for RNA-seq of tumor biopsies and virus assignment were followed according to a previous study from our group (31). Briefly, excess tumor specimens were collected under the 09-156 IRB protocol. Tissue biopsies were flash-frozen and stored in liquid nitrogen until analysis. For RNA extraction, cryopreserved samples were treated overnight with RNAlater at –80°C before proceeding with tissue homogenization using the Qiagen TissueRuptor II. RNA was collected and purified using the QIAGEN AllPrep DNA/RNA Mini kit according to the manufacturer’s instructions. Library preparation and sequencing were performed by Novogene using paired-end sequencing on a NovaSeq6000. Raw sequence reads were aligned and quantified to a decoy-aware human (GRCh38.p13; Ensembl version 102) and MCPyV (R17b; NCBI NC_010277.2) concatenated transcriptome using Salmon (32). Normalized counts were generated using TxImport (33) and DEseq2 (34). Samples were assigned virus positive if transcripts identified for MCPyV Large-T were greater than 100 or MCPyV small T transcripts greater than 10.

### Statistics.

Statistical analyses were conducted using R software (version 4.4.2). Numerical variables were assessed for normality by histograms and the Shapiro-Wilk test. For normally distributed variables, data were summarized using the mean ± SD, and group comparisons were performed using the 2-tailed Student’s *t* test. Nonnormally distributed variables were described with the median and interquartile range (IQR) and group comparisons were conducted using the Wilcoxon rank-sum test. Categorical variables were analyzed using Fisher’s exact or the χ^2^ test for contingency tables. A *P* value less than 0.05 was considered significant.

Univariate analysis was performed using Cox proportional hazards regression to evaluate the relationship between each genetic alteration in the OncoPanel and MCC-SS. Due to the large number of genes tested, the Benjamini-Hochberg method was applied to control for the FDR. Genes with an adjusted *P* < 0.05 after correction were included in multivariable analysis. Multivariable Cox regression was used to assess the effect of genetic alterations on MCC-SS, adjusting for potential confounders such as age, disease stage, use of immunotherapy, and TMB. Multicollinearity among variables was evaluated using variance inflation factors (VIF). The proportional hazards assumption was tested using Schoenfeld residuals to assess tine-dependent effects of covariates. Furthermore, 4 sensitivity analyses were performed to evaluate the robustness of the primary findings: (a) immunotherapy variable was excluded from the multivariable model to mitigate potential immortal time bias; (b) the cohort was restricted to patients with stage I–III disease to minimize the confounding effect of metastatic cases; (c) a subgroup analysis focused on the high-TMB population; and (d) a competing risk analysis was conducted using the Fine-Gray subdistribution hazard model for competing risks to account for non-MCC-related deaths.

### Study approval.

This retrospective study included patients enrolled in the MCC Biobank protocol no. 09-156 at the Dana-Farber Cancer Institute. Patients with available OncoPanel data between 2013 and 2022 were individually assessed for eligibility.

### Data availability.

The underlying data supporting the results of this manuscript are provided in the Supporting Data Values file. The analytic code used in this study is available from the corresponding author upon reasonable request.

## Author contributions

ML, FB, and KK acquired relevant data from patient records. ML and JPDN performed statistical analysis. JLS processed samples for transcriptomic analysis. JLS and AS analyzed transcriptomic and genomic data. ML and JAD wrote the manuscript. MT acquired patient samples. MT, AWS, and JAD assisted in conceptualization and supervision of the study.

## Conflict of interest

The authors have declared that no conflict of interest exists.

## Funding support

This work is the result of NIH funding, in whole or in part, and is subject to the NIH Public Access Policy. Through acceptance of this federal funding, the NIH has been given a right to make the work publicly available in PubMed Central.

- US Public Health Service grants R35CA232128, P01CA203655
- Bridge Project, a partnership between the Koch Institute for Integrative Cancer Research at MIT and the Dana-Farber/Harvard Cancer Center to JAD

## Supplementary Material

Supplemental data

Supplemental tables 1-7

Supporting data values

## Figures and Tables

**Table 1:**
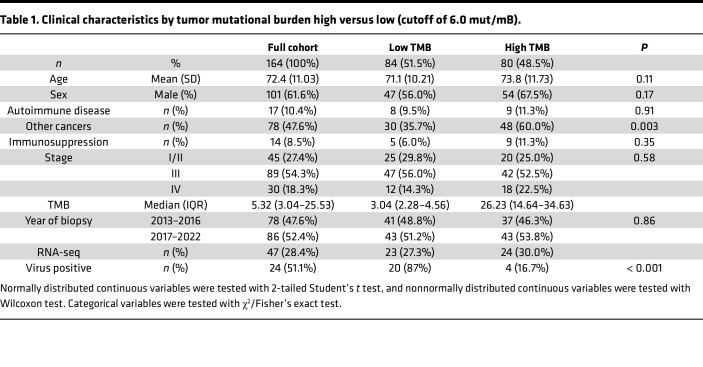
Clinical characteristics by tumor mutational burden high versus low (cutoff of 6.0 mut/mB).

**Table 2:**
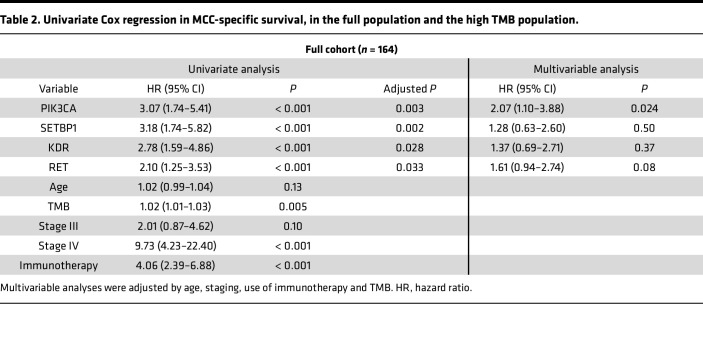
Univariate Cox regression in MCC-specific survival, in the full population and the high TMB population.

**Table 3:**
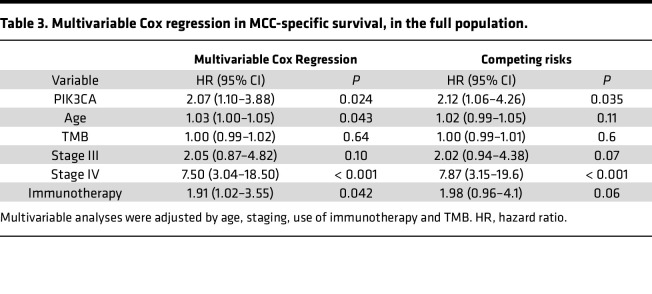
Multivariable Cox regression in MCC-specific survival, in the full population.

## Version 1 04/22/2026

Electronic publication
