# Supplementary material for: High tumor mutational burden and PIK3CA mutations correlate with poor Merkel cell carcinoma–specific survival
Source: JCI Insight. 2026 Apr 22;11(8):e197108. doi: 10.1172/jci.insight.197108 (PMC13135387; doi:10.1172/jci.insight.197108)
Supplement: Supplemental data [file jciinsight-11-197108-s034.pdf]

Supplementary Material 1: Univariate Cox regression for MCC-specific survival in the full population, with adjusted p-values after the Benjamini-Hochberg method.

| Gene | HR (95% CI) | *p-value* | Adjusted *p-value* |
|---|---|---|---|
| *PIK3CA* | 3.07 (1.74 – 5.41) | < 0.001 | 0.003 |
| *SETBP1* | 3.18 (1.74 – 5.82) | < 0.001 | 0.002 |
| *KDR* | 2.78 (1.59 – 4.86) | < 0.001 | 0.028 |
| *RET* | 2.1 (1.25 – 3.53) | 0.005 | 0.033 |
| *NF1* | 2.07 (1.13 – 2.79) | 0.018 | 0.09 |
| *ASXL1* | 2.14 (1.11 – 4.13) | 0.023 | 0.1 |
| *RB1* | 1.68 (1.02 – 2.79) | 0.042 | 0.15 |
| *ARID1B* | 1.88 (1.01 – 3.50) | 0.046 | 0.14 |
| *KMT2D* | 1.73 (1.00 – 3.00) | 0.052 | 0.14 |
| *CREBBP* | 1.84 (0.95 – 3.54) | 0.070 | 0.17 |
| *ARID1a* | 1.75 (0.94 – 3.23) | 0.076 | 0.17 |
| *NOTCH2* | 1.64 (0.88 – 3.05) | 0.12 | 0.25 |
| *TP53* | 1.46 (0.88 – 2.43) | 0.14 | 0.27 |
| *KMT2A* | 1.50 (0.84 – 2.67) | 0.17 | 0.30 |
| *PIK3CB* | 1.38 (0.68 – 2.82) | 0.38 | 0.63 |
| *ROS1* | 1.30 (0.67 – 2.52) | 0.43 | 0.68 |
| *MTOR* | 0.76 (0.38 – 1.55) | 0.46 | 0.67 |
| *ERBB4* | 1.29 (0.63 – 2.62) | 0.49 | 0.68 |
| *APC* | 1.24 (0.68 – 2.26) | 0.49 | 0.64 |
| *BRCA2* | 1.23 (0.68 – 2.21) | 0.49 | 0.61 |
| *SMARCA4* | 0.85 (0.42 – 1.72) | 0.65 | 0.77 |
| *FAT1* | 0.87 (0.41 – 1.83) | 0.71 | 0.81 |
| *CUX1* | 1.11 (0.56 – 2.20) | 0.76 | 0.83 |
| *NOTCH1* | 1.09 (0.63 – 1.87) | 0.77 | 0.8 |
| *GLI2* | 0.97 (0.41 – 2.25) | 0.94 | 0.94 |

Supplementary Material 2: Multivariable Cox regression model for *PIK3CA* and MCC-specific survival, performed separately for each selected gene and adjusted variables.

| Variable | HR (95% CI) | *p* |
|---|---|---|
| *PIK3CA* | 2.07 (1.10 – 3.88) | 0.024 |
| Age | 1.03 (1.00 – 1.05) | 0.043 |
| TMB | 1.00 (0.99 – 1.02) | 0.64 |
| Stage III | 2.05 (0.87 – 4.82) | 0.10 |
| Stage IV | 7.50 (3.04 – 18.50) | < 0.001 |
| Immunotherapy | 1.91 (1.02 – 3.55) | 0.042 |

Supplementary Material 3: Multivariable Cox regression model for SETBP1 and MCC-specific survival, performed separately for each selected gene and adjusted variables.

| Variable | HR (95% CI) | *p* |
|---|---|---|
| *SETBP1* | 1.28 (0.63 – 2.60) | 0.50 |
| Age | 1.02 (0.99 – 1.05) | 0.19 |
| TMB | 1.01 (1.00 – 1.02) | 0.27 |
| Stage III | 1.81 (0.77 – 4.24) | 0.17 |
| Stage IV | 6.37 (2.57 – 15.76) | < 0.001 |
| Immunotherapy | 2.12 (1.15 – 3.92) | 0.017 |

Supplementary Material 4: Multivariable Cox regression model for KDR and MCC-specific survival, performed separately for each selected gene and adjusted variables.

| Variable | HR (95% CI) | *p* |
|---|---|---|
| *KDR* | 1.37 (0.69 – 2.71) | 0.37 |
| Age | 1.02 (0.99 – 1.05) | 0.17 |
| TMB | 1.01 (0.99 - 1.02) | 0.39 |
| Stage III | 1.91 (0.81 – 4.49) | 0.14 |
| Stage IV | 6.37 (2.60 – 15.65) | < 0.001 |
| Immunotherapy | 2.13 (1.16 – 3.93) | 0.015 |

Supplementary Material 5: Multivariable Cox regression model for RET and MCC-specific survival, performed separately for each selected gene and adjusted variables.

| Variable | HR (95% CI) | *p* |
|---|---|---|
| *RET* | 1.61 (0.94 – 2.74) | 0.08 |
| Age | 1.02 (1.00 – 1.05) | 0.07 |
| TMB | 1.01 (0.99 – 1.02) | 0.38 |
| Stage III | 1.86 (0.79 – 4.37) | 0.15 |
| Stage IV | 6.85 (2.81 – 16.71) | < 0.001 |
| Immunotherapy | 2.11 (1.16 – 3.83) | 0.014 |

Supplementary Material 6: Baseline clinicopathologic and treatment characteristics according to *PIK3CA* mutation status. Normally distributed continuous variables were tested with two-tailed Student's t-test and non-normally distributed continuous variables were tested with Wilcoxon test. Categorical variables were tested with chi-square/Fisher exact test.

| Variable | | *PIK3CA* mutated | *PIK3CA* wild type | *p* |
|---|---|---|---|---|
| n | | 22 (27.5%) | 58 (72.5%) | |
| Age | Mean (SD) | 72.6 (16.3) | 74.3 (9.6) | 0.64 |
| Male Sex | n (%) | 12 (54.5%) | 42 (72.4%) | 0.21 |
| Stage | I/II | 6 (27.3%) | 14 (24.1%) | 0.62 |
| | III | 10 (45.5%) | 33 (56.9%) | |
| | IV | 6 (27.3%) | 11 (19.0%) | |
| TMB | Median (IQR) | 36.1 (18.0 – 44.8) | 25.3 (13.5 – 31.0) | 0.04 |
| Immunotherapy | n (%) | 14 (63.6%) | 22 (37.9%) | 0.07 |

Supplementary Material 7. Prognostic impact of *PIK3CA* status across different clinical contexts. Hazard ratios (HR), 95% confidence intervals (CI), and p-values derived from multivariable Cox regression models and sensitivity analyses. The primary multivariable model was adjusted for age, stage, immunotherapy use, and tumor mutational burden (TMB). Sensitivity analyses include: (1) high-TMB subgroup; (2) model excluding immunotherapy; (3) stage I–III restricted cohort; and (4) Fine-Gray subdistribution hazard model for competing risks.

| Sensitivity Analysis | HR (95% CI) | *p* |
|---|---|---|
| Multivariable Cox Regression | 2.07 (1.10 – 3.88) | 0.024 |
| High TMB Population | 2.52 (1.20 – 5.27) | 0.014 |
| Excluding Immunotherapy variable | 2.43 (1.31 – 4.54) | 0.005 |
| Excluding Stage IV patients | 3.03 (1.22 – 7.51) | 0.017 |
| Competing Risks | 2.12 (1.06 – 4.26) | 0.035 |
